# Quantitative trait locus-specific genotype × alcoholism interaction on linkage for evoked electroencephalogram oscillations

**DOI:** 10.1186/1471-2156-6-S1-S123

**Published:** 2005-12-30

**Authors:** Lisa J Martin, Christy L Avery, Jeff T Williams, Kari E North

**Affiliations:** 1 Center for Epidemiology and Biostatistics, Cincinnati Children's Hospital Medical Center, Cincinnati, OH, USA; 2 Department of Epidemiology, University of North Carolina, Chapel Hill, NC, USA; 3 Department of Genetics, Southwest Foundation for Biomedical Research, San Antonio, TX, USA; 4 Southwest National Primate Research Center, Southwest Foundation for Biomedical Research, San Antonio, TX, USA

## Abstract

We explored the evidence for a quantitative trait locus (QTL)-specific genotype × alcoholism interaction for an evoked electroencephalogram theta band oscillation (ERP) phenotype on a region of chromosome 7 in participants of the US Collaborative Study on the Genetics of Alcoholism. Among 901 participants with both genotype and phenotype data available, we performed variance component linkage analysis (SOLAR version 2.1.2) in the full sample and stratified by DSM-III-R and Feighner-definite alcoholism categories. The heritability of the ERP phenotype after adjusting for age and sex effects in the combined sample and in the alcoholism classification sub-groups ranged from 40% to 66%. Linkage on chromosome 7 was identified at 158 cM (LOD = 3.8) in the full sample and at 108 in the non-alcoholic subgroup (LOD = 3.1). Further, we detected QTL-specific genotype × alcoholism interaction at these loci. This work demonstrates the importance of considering the complexity of common complex traits in our search for genes that predispose to alcoholism.

## Background

Neurophysiological features extracted from electroencephalogram (EEG) data, such as event related potentials (ERPs), provide a non-invasive endophenotype to study cognitive functioning in humans [1]. ERPs are complex traits influenced by genes and environment, and plausibly by an interaction between the two. Because familial aggregation of ERPs has been demonstrated [2,3] and because alcoholism is associated with alterations of ERPs [4,5], we assessed the evidence for genotype × alcoholism interaction (G × A) for an ERP for target case frontal theta band and a region on chromosome 7 [6]. The identification of causal genes that underlie common complex diseases represents one of the last great challenges of genomic era [7], and modeling the complexities of these phenotypes has the potential to increase statistical support for linkage, to more precisely localize the quantitative trait loci (QTLs), and thus strengthen our ability to find these genes.

## Methods

### Population

The US Collaborative Study on the Genetics of Alcoholism (COGA) began in 1989 in order to elucidate genetic mechanisms that influence susceptibility to alcohol abuse and related phenotypes [8]. The COGA data set provided for Genetic Analysis Workshop 14 (GAW14) includes 1,350 family members from 143 pedigrees (range 5–32 individuals), from six United States sites. The racial distribution was 66.5% non-Hispanic White, 11.8% non-Hispanic Black, 5.7% Hispanic, 1.7% others, and 14.2% who did not report race/ethnicity. Phenotypic information provided for GAW14 included DSM-III-R alcoholism diagnosis, EEG phenotypes, and age at exam. In addition, microsatellite genotypes spaced at approximately 10-cM intervals across the genome were available.

### ERP phenotypes

Details of the EEG phenotypes have been described elsewhere [6,9]. Briefly, the phenotype of interest is a P300 ERP, elicited through superposition of the delta (1–3 Hz) and theta (3–7 Hz) band oscillatory responses, where mean event related energy response is calculated via the S-transformation [10]. Data were extracted from a time-frequency region of interest (TFROI-P300 time window), and averaged across recording channels in three regions.

### Alcoholism classification

Alcoholism was diagnosed using the Diagnostic and Statistical Manual of Mental Disorders, Third Edition, Revised (DSM-III-R) criteria for alcohol dependence and the Feighner et al. [11] criteria for definite alcoholism. According to these criteria, we defined three groups for analysis. The full sample comprised all individuals with data on the ERP phenotype (*n *= 901). The "affected" (alcoholic) sample comprised those individuals who displayed at least one symptom in three out of four possible categories of symptoms (*n *= 457). The "unaffected" (not alcoholic) sample comprised individuals who used alcohol but did not report any symptoms of alcohol dependence (*n *= 148). There is no overlap between affected and unaffected classifications. Individuals who failed to be classified as affected or unaffected (*n *= 296) were considered unknown because clinically no definite diagnosis could be made. These individuals are likely a heterogeneous group, which would impair our ability to detect gene × environment interactions.

### Analytical methods

Univariate quantitative genetic analysis was performed to partition the phenotypic variance of the ERP into its additive genetic and environmental variance components using maximum likelihood variance decomposition methods [12] implemented in SOLAR. All analyses were adjusted for the fixed effects of sex, age, age by sex interaction, age^2^, and age^2 ^by sex interaction on the phenotype trait mean. Because data were adjusted for covariates, heritability reported will be the proportion of additive genetic variance over the phenotypic variance after adjustment for covariates. Additionally, a correction for ascertainment was made by conditioning the likelihood of each pedigree on the phenotypic values of its probands [13]. A genome scan was implemented to confirm the linkage previously reported by Jones et al. [6]. The *t*-distribution function was implemented to account for the slightly kurtotic distribution of the phenotype [14]. Marker allele frequencies were derived from pedigree founders and multipoint IBD sharing was estimated using SOLAR.

### Alcoholism effects

To examine the effect of alcoholism on the phenotype of interest, we used four methods: inclusion of alcoholism as a covariate (0 = unaffected, 1 = affected), test for additive G × A interaction, linkage analysis by subgroup, and test for G × A at the linkage peak.

To test for evidence of G × A, we extended the expected genetic covariance to have different effects based on environment:

COV(G_e1_,G_e2_) = 2 φ ρ_G_(_e1,e2_) σ_ge1 _σ_ge2_,

where φ is the coefficient of kinship between the two individuals, ρ_G_(_e1,e2_) is the genetic correlation between the trait in the two environments, and σ_ge1 _and σ_ge2 _are the genetic standard deviations in the two environments (affected and unaffected) [15-17]. In the absence of G × A, the genetic correlation between relatives for a trait should be one (H_o_: ρ_G_(_e1,e2_) = 1.0) and the genetic variances in the two groups should be equal (H_o_: σ_ge1 _= σ_ge2_). Conversely, if there is G × A interaction, the genetic correlation between the groups will be significantly less than one (H_A_: ρ_G_(_e1,e2_) < 1.0) and/or the genetic variances will not be equal between the groups (H_A_: σ_ge1 _≠ σ_ge2_). Rejection of either null hypothesis is evidence of an additive G × A. To formally test these hypotheses, we used the likelihood ratio test to compare restricted models in which ρ_G _is constrained to one or the genetic standard deviations are constrained to be equal. When comparing models with standard deviations constrained to be equal, interpretation of significant differences were based on the assumption of an asymptotic  distribution for the likelihood test statistic. However, for the model that restricted the genetic correlation to one, the genetic correlation was constrained to the upper boundary of the parameter space (ρ_G _= 1.0), thus the test statistic is as a 1/2:1/2 mixture of a  distribution and a point mass at zero [18].

To test for evidence of a QTL-specific G × A, two additional parameters were added, the marker specific standard deviations for the affecteds and unaffecteds [19]. Therefore, the expected genetic covariance between a pair of relatives is defined as

COV(G_e1_,G_e2_) = 2 φ ρ_G_(_e1,e2_) σ_g e1 _σ_ge2 _+π_q_σ_qe1 _σ_qe2_,

where π_q _provides the probability that individuals are identical by descent at a QTL, which is linked to a genetic marker locus, and σ_qe1 _and σ_qe2 _are the marker specific genetic standard deviations for the two environments. If there is QTL specific G × A, then the marker-specific genetic standard deviations will be significantly different from each other.

## Results

The heritability of the ERP phenotype was estimated at 40% in the full sample and ranged from 40% to 66%, with unaffected individuals displaying the highest heritability (Table 1). However, it is important to note that the standard errors of the heritability for the unaffected group overlapped with the heritability estimate of both the full sample and the alcoholic sample, suggesting that the increase is not statistically significant. The variance explained by covariate effects was similar across groups, varying from 5% to 16%. Interestingly, no covariate effect of alcoholism classification was detected.

**Figure 1:**
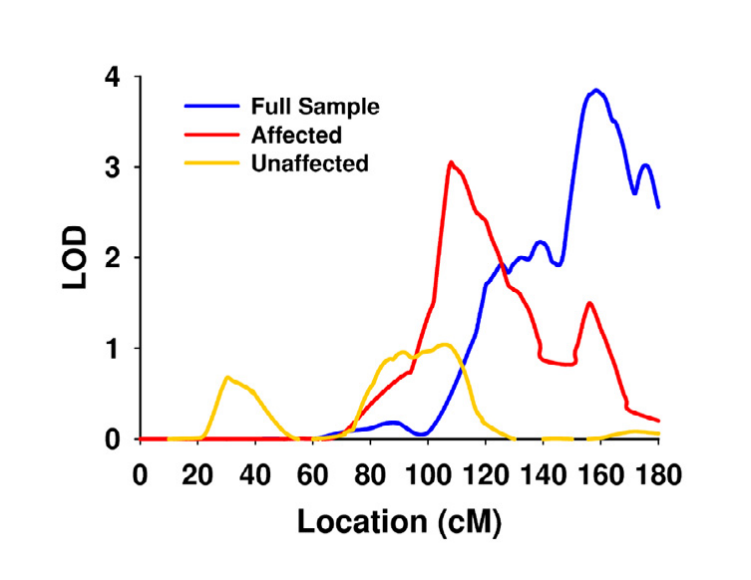
Multipoint LOD scores on chromosome seven in the full dataset and by alcoholism classification.

In a preliminary genome scan we observed a maximum LOD score of 3.8 on chromosome 7 at 158 cM in the full sample. The results from linkage analyses conducted in the full and alcoholism classification sub-group samples are displayed in Figure 1. These results suggest that alcoholism status influences both the magnitude and location of the linkage evidence, with the maximum LOD score shifting 50 cM towards the *p*-terminus.

Although no additive G × A was detected, significant QTL-specific G × A was detected when comparing affected participants to unaffected participants for both loci. Although the subset linkage analysis by alcoholism suggests that there would be a stronger effect in alcoholics as that group had the greatest linkage signal, the marker specific standard deviations for the unaffected group were larger than the affected group at locus 108 (*p *= 0.0055) and 158 cM (*p *= 0.012), suggesting a stronger genetic effect in the unaffected group.

## Discussion

The purpose of this paper was to examine the effects of alcoholism on an ERP phenotype on a region of chromosome 7, because this phenotype has been associated with alcoholism previously. Although we failed to identify a linear effect of alcoholism (covariate modeling), both the linkage data from alcoholism subsets and formal tests of QTL-specific G × A support differential genetic effects in alcoholic and non-alcoholic individuals.

The heritability of ERP phenotype in the full sample and in the alcoholism classification sub-groups ranged from 40% to 66%, but these differences were not statistically different because the standard errors of the estimates overlapped. When subsetting based on alcoholism, we found that alcoholic and non-alcoholic participants displayed a peak LOD score 50 cM toward the *p*-terminal end. It is unclear which factors are influencing this shift; possibilities include low information content of the markers between these two linkage peaks or two QTLs. However, an oligogenic linkage analysis did not support the presence of a second QTL on chromosome 7 (data not shown).

Significant QTL-specific G × A interaction was detected when comparing affected participants to unaffected participants at 108 and 158 cM. When estimating QTL specific G × A, a total of 605 individuals entered the analysis, representing 1,108 relative pairs, including 404 relative pairs discordant for alcoholism, 640 relative pairs concordant for alcoholism and 64 relative pairs concordant for non-alcoholism status. When compared to alcoholics, non-alcoholics displayed larger marker-specific standard deviations at 108 and 158 cM. This may seem counterintuitive because the non-alcoholics displayed lower evidence of linkage (LODs ≤ 1.0). However, given the small number of relative pairs concordant for unaffected status, the power to detect linkage in this group was low. Indeed, simulation analysis suggests that the LOD was underestimated by 15% while for affected, the LOD was overestimated by 25%. However, by formally modeling a genotype × environment interaction, we are able to include the 404 pairs of relatives discordant for alcoholism and thus improve our ability to detect genetic effects.

We also explored the effect of alcoholism classification, by examining the effect of inclusion of the individuals who were not clearly affected or unaffected (data not shown). We found that including these ambiguous individuals eliminated our ability to detect G × A. There are several possible reasons for this such as genetic or environmental heterogeneity of the ambiguous group. Additionally, we examined only the White sample to remove race/ethnicity effects, and found that the G × A interaction at 108 and 158 cM persisted.

## Conclusion

In summary, using the COGA data, we attempted to disentangle the effects of alcoholism on an EEG theta band oscillation phenotype that has been linked to a region of chromosome 7. Although we failed to detect evidence for an additive G × A, we detected evidence for a different magnitude of effect in alcoholics versus non-alcoholics at the QTL level. Further, the subsetting of this data shifted the linkage peak 50 cM *p*-ter, suggesting a second QTL or poor localization of the trait. This work demonstrates the importance of considering the interaction of genes and environment in the etiology of common complex traits. In future studies, researchers must consider the impact of genetic and environmental heterogeneity and formally test for these effects to improve our ability to find and localize genes involved in complex traits such as alcoholism.

## Abbreviations

COGA: Collaborative Study on the Genetics of Alcoholism

EEG: Electroencephalogram

ERP: Event-related potential

G × A: Genotype × alcoholism

GAW14: Genetic Analysis Workshop 14

QTL: Quantitative trait loci

## Authors' contributions

LJM and KEN performed statistical analyses and interpreted results. JTW and CLA assisted in the interpretation of the results. All authors read and approved the final manuscript.

## Figures and Tables

**Table 1:** Model parameter estimates in the combined and alcoholism classification sub-groups

	Number of individuals	Number of relative pairs	Heritability	Variance explained by covariates
Full sample	901	2498	0.40 ± 0.07	0.16
Affected	457	640	0.41 ± 0.11	0.15
Unaffected	148	64	0.66 ± 0.28	0.05
